# CRISPR/Cas9-Constructed Pseudorabies Virus Mutants Reveal the Importance of UL13 in Alphaherpesvirus Escape from Genome Silencing

**DOI:** 10.1128/JVI.02286-20

**Published:** 2021-02-24

**Authors:** Jolien Van Cleemput, Orkide O. Koyuncu, Kathlyn Laval, Esteban A. Engel, Lynn W. Enquist

**Affiliations:** a Department of Molecular Biology, Princeton University, Princeton, New Jersey, USA; b Princeton Neuroscience Institute, Princeton University, Princeton, New Jersey, USA; Northwestern University

**Keywords:** herpesvirus, alphaherpesvirus, pseudorabies virus, latency, PNS neurons

## Abstract

Latent and recurrent productive infection of long-living cells, such as neurons, enables alphaherpesviruses to persist in their host populations. Still, the viral factors involved in these events remain largely obscure. Using a complementation assay in compartmented primary peripheral nervous system (PNS) neuronal cultures, we previously reported that productive replication of axonally delivered genomes is facilitated by pseudorabies virus (PRV) tegument proteins. Here, we sought to unravel the role of tegument protein UL13 in this escape from silencing. We first constructed four new PRV mutants in the virulent Becker strain using CRISPR/Cas9-mediated gene replacement: (i) PRV Becker defective for UL13 expression (PRV ΔUL13), (ii) PRV where UL13 is fused to eGFP (PRV UL13-eGFP), and two control viruses (iii and iv) PRV where VP16 is fused with mTurquoise at either the N terminus (PRV mTurq-VP16) or the C terminus (PRV VP16-mTurq). Live-cell imaging of PRV capsids showed efficient retrograde transport after axonal infection with PRV UL13-eGFP, although we did not detect dual-color particles. However, immunofluorescence staining of particles in mid-axons indicated that UL13 might be cotransported with PRV capsids in PNS axons. Superinfecting nerve cell bodies with UV-inactivated PRV ΔUL13 failed to efficiently promote escape from genome silencing compared to UV-PRV wild type and UV-PRV UL13-eGFP superinfection. However, UL13 does not act directly in the escape from genome silencing, as adeno-associated virus (AAV)-mediated UL13 expression in neuronal cell bodies was not sufficient to provoke escape from genome silencing. Based on this, we suggest that UL13 may contribute to initiation of productive infection through phosphorylation of other tegument proteins.

**IMPORTANCE** Alphaherpesviruses have mastered various strategies to persist in an immunocompetent host, including the induction of latency and reactivation in peripheral nervous system (PNS) ganglia. We recently discovered that the molecular mechanism underlying escape from latency by the alphaherpesvirus pseudorabies virus (PRV) relies on a structural viral tegument protein. This study aimed at unravelling the role of tegument protein UL13 in PRV escape from latency. First, we confirmed the use of CRISPR/Cas9-mediated gene replacement as a versatile tool to modify the PRV genome. Next, we used our new set of viral mutants and AAV vectors to conclude the indirect role of UL13 in PRV escape from latency in primary neurons, along with its spatial localization during retrograde capsid transport in axons. Based on these findings, we speculate that UL13 phosphorylates one or more tegument proteins, thereby priming these putative proteins to induce escape from genome silencing.

## Abstract (precis)

Alphaherpesviruses have mastered various strategies to persist in an immunocompetent host, including the induction of latency and reactivation in peripheral nervous system (PNS) ganglia. We recently discovered that the molecular mechanism underlying escape from latency by the alphaherpesvirus pseudorabies virus (PRV) relies on a structural viral tegument protein.

## INTRODUCTION

Alphaherpesviruses, including herpes simplex virus (HSV-1 and -2), varicella zoster virus (VZV), and pseudorabies virus (PRV), are ancient pathogens that have evolved with their hosts. Over the years, these viruses have developed a remarkable way to persist in the host population: a lifelong persistent infection, termed latency or quiescence, with periodic reactivation, in which the viral genome reenters productive replication (1). Productive replication occurs after a cascade-dependent process of viral gene transcription followed by assembly of new progeny virions (2). Productive replication is often accompanied by pathologies such as oral and genital ulcerations, dermatomal rash with pain and itching, reproductive disorders like abortion and neonatal disease, or nervous system disorders such as encephalitis (3). As far as is known, no pathologies are directly associated with the latent infection. A hallmark of all alphaherpesviruses is the establishment of latency in peripheral ganglia of the nervous system (e.g., the trigeminal ganglion) after primary productive infection in mucosal epithelia. Remarkably, infected neurons do not die, but rather the viral genome is stably retained in neuronal nuclei in the absence of detectable viral protein expression, rendering the infection undetectable by the immune system. However, periodic reactivation and subsequent progeny virion production is essential for efficient transmission of infection to new hosts. Reactivated virions travel back from neuronal cell bodies to mucosae via anterograde axonal transport. Efficient replication in the epithelium promotes shedding of infectious progeny virions in mucosal secretions, which are available to infect new hosts (1–3).

Despite the crucial role of latency and periodic reactivation in alphaherpesvirus persistence and pathogenesis, the molecular mechanisms underlying these events are not well understood. Koyuncu et al. (4) recently identified two different mechanisms for pseudorabies virus (PRV), a swine alphaherpesvirus closely related to HSV-1 and -2, to escape from latency: (i) a cellular stress-mediated slow route and (ii) a viral tegument-mediated fast route. The former route involves cellular protein kinase A and c-Jun N-terminal kinase activity, and has already extensively been studied, while the latter acts independently from cellular kinases but the mechanism so far remains obscure. These findings were discovered using an *in vitro* trichamber model, in which peripheral neuronal cell bodies are physically and fluidically separated from their axonal termini (5). Administration of a low viral dose in the axonal compartment mimics the natural route of neuronal invasion by long-distance retrograde axonal transport. This protocol results in the establishment of a quiescent, reactivatable infection in neuron cell bodies (6). Interestingly, simultaneous delivery of a high dose of UV-inactivated or nucleocapsid-deficient PRV particles to nerve cell bodies enabled infectious PRV genomes to escape from latency after inoculation at the axonal compartment (4). These results implied that specific viral tegument proteins are required to induce a productive viral infection in neurons. The researchers ruled out the PRV early protein EP0 and the viral kinase Us3 (4). However, the exact viral tegument protein or proteins responsible for PRV escape from genome silencing remain unknown. In this context, HSV-1 tegument protein UL13 is known to promote viral transcription through alterations in RNA polymerase II phosphorylation (7). UL13 is a serine/threonine kinase conserved throughout all members of the *Herpesviridae* (8). Alternatively, this viral kinase might activate specific cellular pathways or phosphorylate other viral tegument proteins, which in turn may stimulate viral gene transcription.

The main objective of our study was to pinpoint the role of UL13 in the escape from PRV genome silencing. Therefore, we constructed a PRV mutant that did not express UL13, and an adeno-associated virus (AAV) vector expressing UL13 to determine if UL13 was necessary and sufficient to induce PRV escape from genome silencing in neurons. In addition, we hypothesized that upon entry of PRV virions into axons, nucleocapsids harboring the viral genome are transported separately from viral tegument proteins that orchestrate the onset of viral genome transcription. To test this hypothesis, we sought to track UL13 protein transport in axons along with nucleocapsids. Therefore, we also constructed PRV mutants harboring fluorescently tagged UL13 or VP16 to track viral protein transport in axons via live-cell imaging. Tegument protein VP16 is a viral transcription activator that is transported separately from PRV nucleocapsids in chicken embryo dorsal root ganglia (DRG) (9). All PRV mutants were constructed using CRISPR/Cas9, one of the most powerful and versatile tools for precise gene editing at this time (10). The traditional approach to construct herpesvirus mutants involved homologous recombination of DNA fragments with the viral genome, often introduced as a bacterial artificial chromosome (BAC), which is a slow and laborious process. Genome engineering with CRISPR/Cas9 relies on a single guide RNA (sgRNA) that directs an endonuclease (Cas9) toward a specific gene locus due to sequence homology. The DNA at this locus is cleaved and subsequently “repaired” by mammalian DNA repair mechanisms that are inherently error-prone, thereby inducing insertions, deletions and mutations at the target site, potentially knocking out expression of the specific gene product. Alternatively, foreign genes (e.g., fluorophore genes) can be knocked-in through homologous recombination in the presence of a DNA donor with homology arms. Using this method, fluorophore-tagged viruses can be produced to facilitate screening. The CRISPR/Cas9 toolbox has already been exploited for its gene editing potential in herpesviruses, including herpes simplex virus (HSV), PRV, and cytomegalovirus (CMV) (11–14). However, the technology of editing functional PRV genes is still in development.

We first confirmed the use of CRISPR/Cas9 as an effective method to modify the PRV genome. Following characterization of this newly constructed set of mutants, we confirmed via live-cell imaging that VP16 is not cotransported with PRV nucleocapsids during retrograde axonal transport upon viral entry into axons. The fluorescent signal of UL13-eGFP was too faint for live-cell imaging, but immunofluorescence staining indicated that UL13 is cotransported with PRV nucleocapsids during retrograde axonal transport. Finally, we demonstrated that UL13 is indirectly involved in PRV genome escape from silencing.

## RESULTS

### CRISPR/Cas9 is an effective tool to edit PRV genomes.

**FIG 1:**
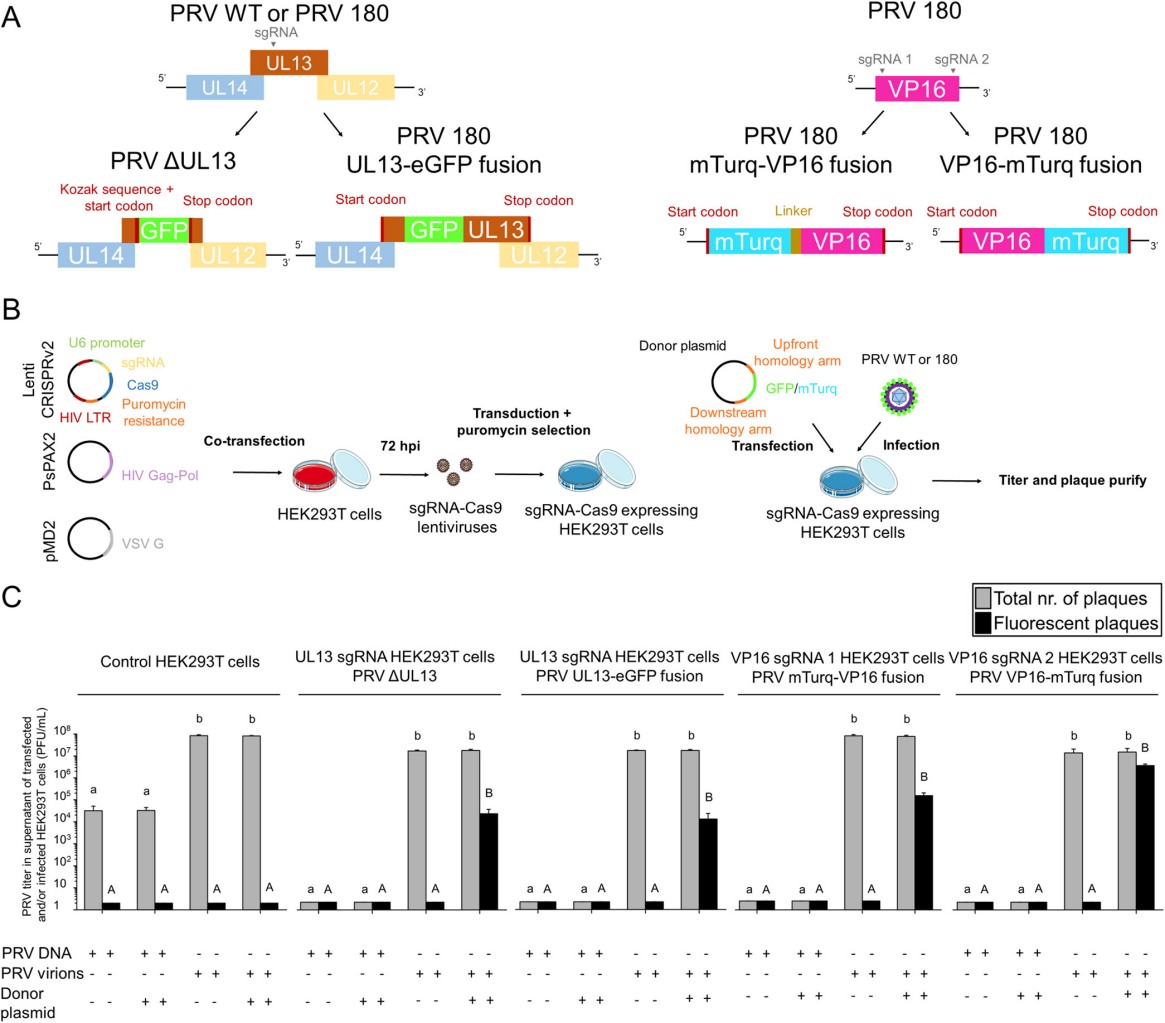
CRISPR/Cas9-mediated mutagenesis of PRV through homology-directed recombineering of the PRV genome. (A) Schematic of UL13 and VP16 targeting and eGFP or mTurq incorporation. (B) Workflow of PRV mutant production. (C) Efficacy of (fluorescent) progeny virus propagation in HEK293T cells with or without stable expression of sgRNA-Cas9 upon transfection of the whole PRV genome, inoculation by PRV virions, and/or addition of donor plasmids. Three independent experiments were performed, and data are represented as means + standard deviations (SD). Different lower case letters indicate significant (*P* < 0.05) differences in the total number of plaques among different experimental conditions. Different upper case letters indicate significant (*P* < 0.05) differences in the number of fluorescent plaques among different experimental conditions.

Different PRV mutants were constructed using CRISPR/Cas9-mediated homologous recombination, as shown schematically in Fig. 1A and B.

The PRV null for UL13 protein expression (PRV ΔUL13) strain was constructed from a PRV wild type (WT) Becker strain background by replacing the middle part of the UL13 coding sequence (CDS) with the eGFP coding sequence. Both ends of the UL13 CDS were left intact, as they overlap those of UL12 and UL14. A UL13-eGFP fusion mutant was constructed in a PRV 180 background. PRV 180 is a virus previously made to express red-fluorescent capsid to allow for imaging of individual viral particles in living neurons (15, 16). Enhanced GFP (eGFP) was inserted at position 105 of UL13, as it could not be fused to the UL13 N or C terminus without polar effects on UL12 and UL14. Both PRV UL13 mutants were produced in a HEK293T cell line expressing Cas9 and UL13 sgRNA (Table 1) targeting the inner region of UL13.

**TABLE 1:** CRISPR/Cas9 target sequences

Name	spCas9 sgRNA + PAM^*a*^	Target sequences (GenBank JF797219.1)
UL13 sgRNA	cgaggccgtcatgacgctgc TGG	77651–77673
VP16 sgRNA 1	gtgcgtggtcgcgttcgacg AGG	9978–10000
VP16 sgRNA 2	acatccggttgagcgcgtcg CGG	11172–11194

a PAM, protospacer adjustment motif; sgRNA, single guide RNA. Lowercase letters represent the spCas9 sgRNA, and uppercase letters are the PAM sequences.

VP16 is abundantly present in the PRV tegument and has already been visualized during transport in axons (9). Accordingly, we fused PRV 180 VP16 to the fluorophore mTurquoise version 2 (mTurq) to serve as an internal control for transport. The coding sequence of VP16 and its flanking regions do not overlap those of other proteins and, thus, mTurquoise v2 could be linked to either the N (mTurq-VP16 PRV180) or C terminus (VP16-mTurq PRV180) of VP16. The former and latter were constructed in HEK293T monoclonal cell lines stably expressing Cas9 and VP16 sgRNA1 or sgRNA2, respectively (Fig. 1A).

First, we assessed the efficacy of viral mutant production using homologous recombination induced by CRISPR/Cas9 by comparing the amount of fluorescent virus produced in control HEK293T cells with monoclonal HEK293T cell lines expressing different sgRNAs and Cas9 (Fig. 1C). Although transfection of 10 μg of whole PRV genomes in control HEK293T cells resulted in production of 10^3^ PFU/ml progeny virus, sgRNA/Cas9-expressing HEK293T cells did not support any detectable viral replication upon transfection of viral DNA (<10 PFU/ml). In contrast, infection with intact PRV virions (multiplicity of infection [MOI] of 1) consistently resulted in efficient viral progeny production with titers ranging from 10^6^ to 10^7^ PFU/ml in all different cell types. In the absence of donor plasmids containing the fluorophore flanked by homology arms, no fluorescent plaques were observed upon assay of progeny virus. However, addition of donor plasmids, followed by PRV inoculation, resulted in the production of fluorophore-harboring progeny viruses with the highest efficacy (>80% of fluorescent plaques) observed for the VP16-mTurq fusion mutant. These results show that CRISPR/Cas9 is a simple and effective way to produce PRV mutants.

### Confirmation of CRISPR/Cas9-constructed PRV mutants.

Mutations were confirmed through PCR amplification followed by Sanger sequencing, immunofluorescence (staining) and Western blot analysis.

**FIG 2:**
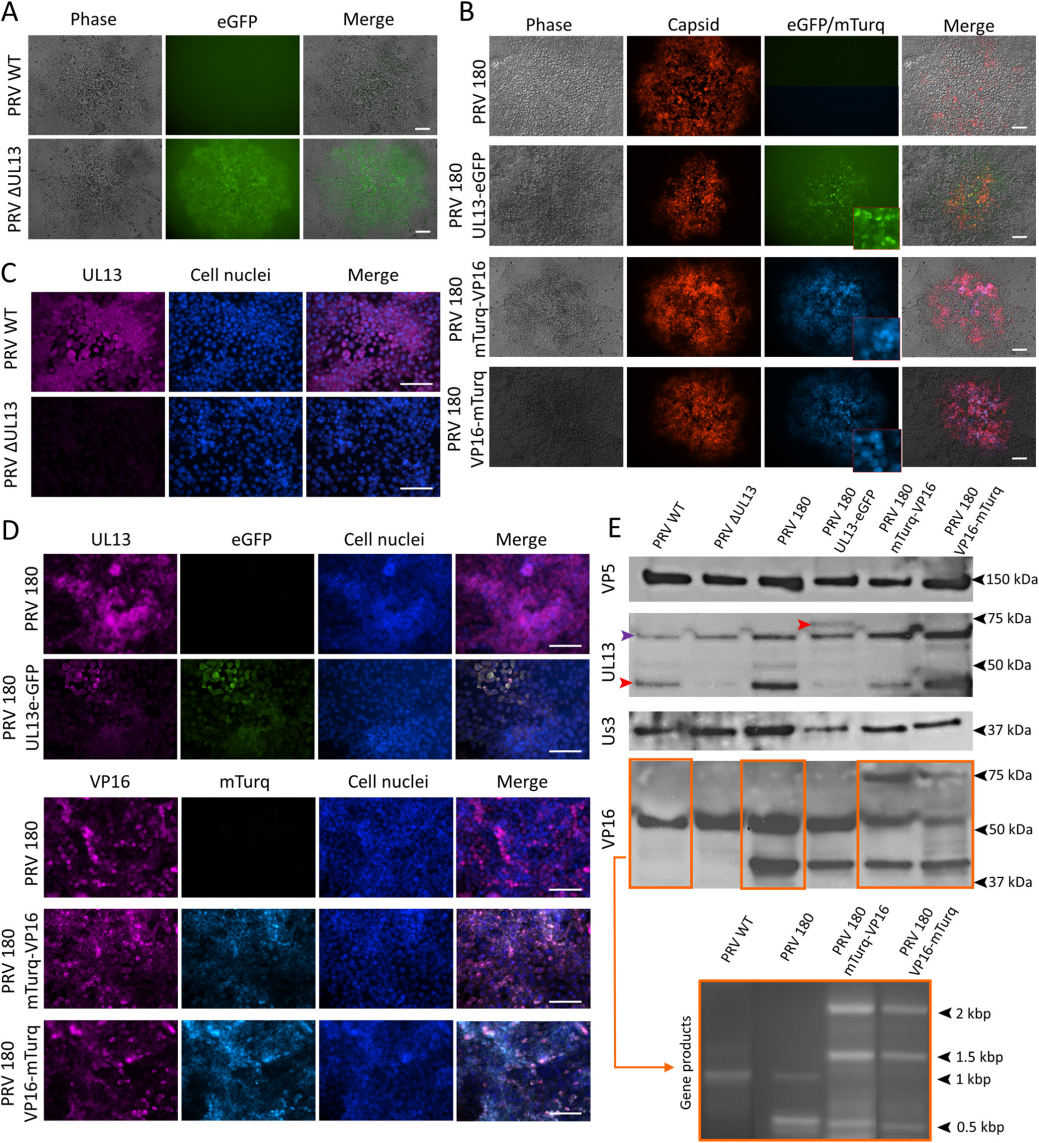
Characterization of newly constructed PRV mutants. All scale bars represent 100 μm. (A) Fluorescence microscopy images of PRV wild type (WT) and PRV ΔUL13 plaques in PK15 cells. (B) Fluorescence microscopy images of PRV 180, PRV 180 UL13-eGFP, PRV 180 mTurq-VP16, and PRV 180 VP16-mTurq plaques in PK15 cells. Magnified images (3×) of the green or blue channels are shown in the lower right corner of the respective images. (C) Absence of UL13 in PRV ΔUL13 was confirmed by immunofluorescence staining using polyclonal rabbit anti-UL13 antibodies. (D) Colocalization of eGFP with UL13 and mTurq with VP16 in PRV 180 UL13-eGFP and PRV 180 mTurq-VP16 or PRV 180 VP16-mTurq, respectively, as confirmed by immunofluorescence staining using polyclonal rabbit anti-UL13 or -VP16 antibodies. (E) Western blot analysis of purified progeny virions (upper image). Agarose gel electrophoresis of VP16 gene PCR products of different PRV strains (lower image).

First, correct insertion of fluorescent protein genes into the genome of plaque-purified PRV mutants was analyzed. DNA of purified virus stocks was first subjected to PCR using region-specific primers (Table 2). Identities were then confirmed by Sanger sequencing. Next, fluorophore expression was evaluated after infection with different PRV mutants. Infection of PK15 cells with PRV ΔUL13 led to significant green fluorescence compared to infection with PRV WT (Fig. 2A). PK15 cells infected with double-tagged PRV 180 UL13-eGFP emitted green fluorescence in addition to red fluorescence, while PRV 180-infected PK15 cells only emitted red fluorescence (Fig. 2B). Interestingly, UL13-eGFP mainly accumulated around the nucleus (perinuclear region) of PK15 cells, as depicted by the magnified image in the lower right corner of Fig. 2B. Likewise, double-tagged PRV 180 mTurq-VP16 and PRV 180 VP16-mTurq induced the formation of double cyan and red fluorescent viral plaques. Viral plaque assays consistently showed that all viral mutant plaques emitted the correct fluorescent signals, showing there was no contamination with parental viral strains.

**TABLE 2:** Primers for qPCR and sequencing

Name	Forward (5′–3′)	Reverse (5′–3′)
UL13 seq	gacgacgcggccgcgctcgacgaggac	ctcgacgagcaggtcgtgcacgtac
VP16 seq	ggacgagagcacccccgggcggaag	ccgcgtcgctcatggtggtcgctg
UL54 qPCR	tgcagctacaccctcgtcc	tcaaaacaggtggttgcagtaaa

Correct protein expression of different PRV mutants was confirmed by double immunofluorescence staining of PK15 cells infected with different PRV viruses, as shown in Fig. 2C and D. PRV WT-infected PK15 cells clearly expressed UL13, while PRV ΔUL13-infected cells did not (Fig. 2C). In Fig. 2D, colocalization analysis in ImageJ of PRV fusion mutants shows perfect overlap between UL13 and eGFP in PRV UL13-eGFP (upper panels) and between VP16 and mTurquoise in PRV 180 mTurq-VP16 and PRV 180 VP16-mTurq (lower panels).

Western blot analysis of purified extracellular virions (10^12^ genome copies) was performed to assess structural incorporation of different proteins into PRV virions. Staining of major capsid protein VP5 functioned as a loading control to correlate protein expression to the amount of capsid protein. Analysis of UL13 demonstrated the absence of UL13 in PRV ΔUL13, compared to PRV WT (red arrow). As expected, the UL13-positive band of PRV UL13-GFP had shifted to ±75 kDa from ± 40 kDa for PRV 180 due to fusion with eGFP (± 27 kDa). The purple arrows indicate a nonspecific band present in all purified PRV strains. Absence of UL13 kinase activity could result in excessive incorporation of another important viral kinase, Us3. However, equal relative amounts of Us3 were present between PRV WT and PRV ΔUL13 strains when normalized to the major capsid protein VP5. While VP16 expressed from PRV WT and from PRV ΔUL13 was present as a single protein band, VP16 of PRV 180 and PRV 180 UL13-eGFP was present as a double band. This intriguing phenomenon was also apparent on gel electrophoresis of PCR-amplified products of the VP16 region of the respective viruses (Fig. 2E and primers are shown in Table 2). This suggests that during production of PRV 180, mutations such as duplications occurred in the PRV 180 genome, resulting in multiple gene transcripts and thus protein bands. For VP16 of PRV 180 VP16-mTurq and PRV 180 mTurq-VP16 virions, a triple band was documented. The upper band of ±75 kDa corresponds to the fusion protein consisting of VP16 (± 50 kDa) and mTurquoise (± 27 kDa).

### Characterization of CRISPR/Cas9-constructed PRV mutant infection in PK15 cells and dissociated superior cervical-ganglion (SCG) neurons.

**FIG 3:**
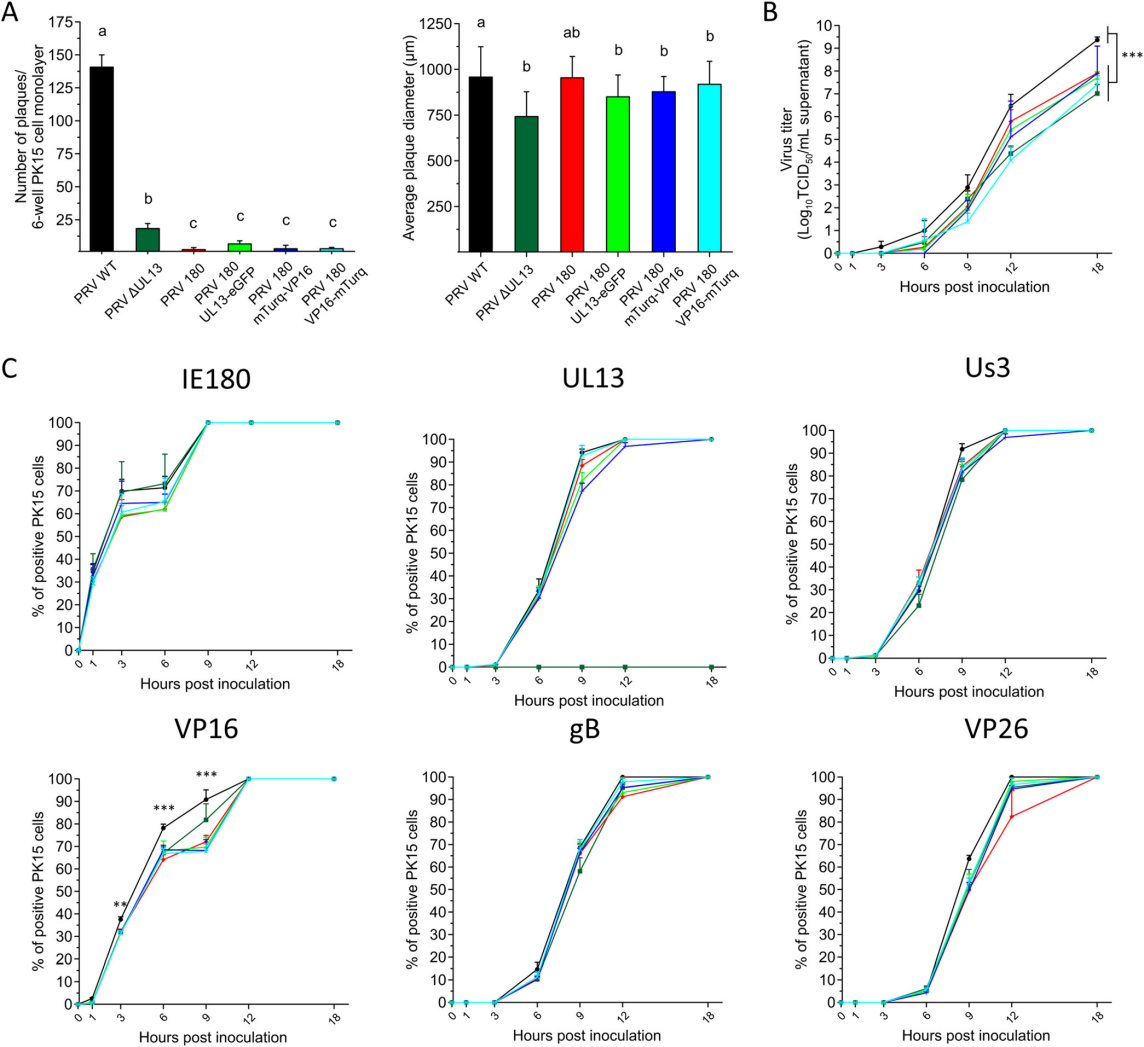
Viral infectivity, spread, and propagation parameters of newly constructed PRV mutants in PK15 cells. (A) Total number of plaques (left) and average plaque diameter (right) were determined 48 hpi upon standardizing the inoculum at 10^6^ genome copies. Experiments were performed in triplicate. Data are represented as means + SD and different lower case letters indicate significant (*P* < 0.05) differences between different viral mutants. (B) Temporal virus propagation was determined on PK15 cells by standardizing the inoculum at an MOI of 1. Significant differences are indicated by asterisks: ***, *P* < 0.001. (C) Expression kinetics of different viral proteins were determined on PK15 cells by immunofluorescence staining using antibodies against IE180 (immediate early protein), UL13 (viral kinase and tegument protein), Us3 (viral kinase and tegument protein), VP16 (tegument protein), gB (envelope protein), and VP26 (capsid protein). Experiments were performed in triplicate. Significant differences are indicated by asterisks: **, *P* < 0.01; ***, *P* < 0.001.

Virus plaque formation was assessed in PK15 cells by standardizing the inoculum at 10^6^ genome copy numbers per PRV mutant (Fig. 3A). Deletion of UL13 in PRV WT resulted in a significant reduction of 82.7 ± 0.3% in viral plaque formation, showing that PRV ΔUL13 is less capable of infecting PK15 cells. In addition, the average size of PRV ΔUL13 plaques was significantly smaller (742 ± 135 μm) compared to that of PRV WT (958 ± 166 μm), pinpointing the importance of UL13 in viral spread. Although in a plaque assay comparing stocks with equivalent numbers of genomes, PRV 180 formed significantly fewer plaques compared to PRV WT, no significant difference was observed in the number of plaques or plaque diameters between PK15 cells inoculated with different PRV 180 or different PRV 180 fusion mutants. This indicates that UL13 or VP16 fusion proteins do not interfere with the ability of PRV virions to infect PK15 cells.

Kinetics of viral protein expression and virus propagation were assessed in PK15 cells by standardizing the inoculum at an MOI of 1. As shown in the Fig. 3B, all viral mutants grew less well compared to PRV WT, as shown in the reduction of virus titers. However, between PRV 180 and different PRV 180 fusion mutants, no overall significant difference was observed in viral titers. Temporal expression of IE180, Us3, gB, or VP26 did not significantly differ between the parental strains and different viral mutants (Fig. 3C). Except for PRV ΔUL13, which lacked UL13 expression, all viruses started expressing UL13 at similar time points. The number of cells expressing VP16 did not differ between parental strains (PRV WT and PRV 180) and their derivatives (PRV ΔUL13 and PRV 180 UL13-eGFP, PRV 180 mTurq-VP16, or PRV 180 VP16-mTurq, respectively). However, all PRV 180 strains had a lower percentage of VP16-positive cells between 3 and 9 h postinfection (hpi) compared to PRV WT and PRV ΔUL13, which might reflect mutations in the genome of PRV 180, as suggested by the multiple VP16 transcripts and proteins expressed by PRV 180 compared to PRV WT. Finally, we evaluated infection of dissociated superior cervical ganglia (SCG) neurons by different PRV mutants. No significant difference was observed among different viruses upon infection at MOI 1. Indeed, all neurons were positive for IE180 at 3 hpi and at 9 hpi, and all neurons expressed PRV late proteins UL13, VP16, and VP26. Still, no virus replication was observed in dissociated SCG neurons, as viral titers in cell supernatant remained undetectable.

Since there were no significant differences in the different infectivity parameters between N- or C-terminal tagged mTurq-labeled VP16 PRV 180 mutants, further experiments were conducted with the N-terminal variant only.

### UL13, but not VP16, is cotransported with PRV capsids in SCG neurons.

**FIG 4:**
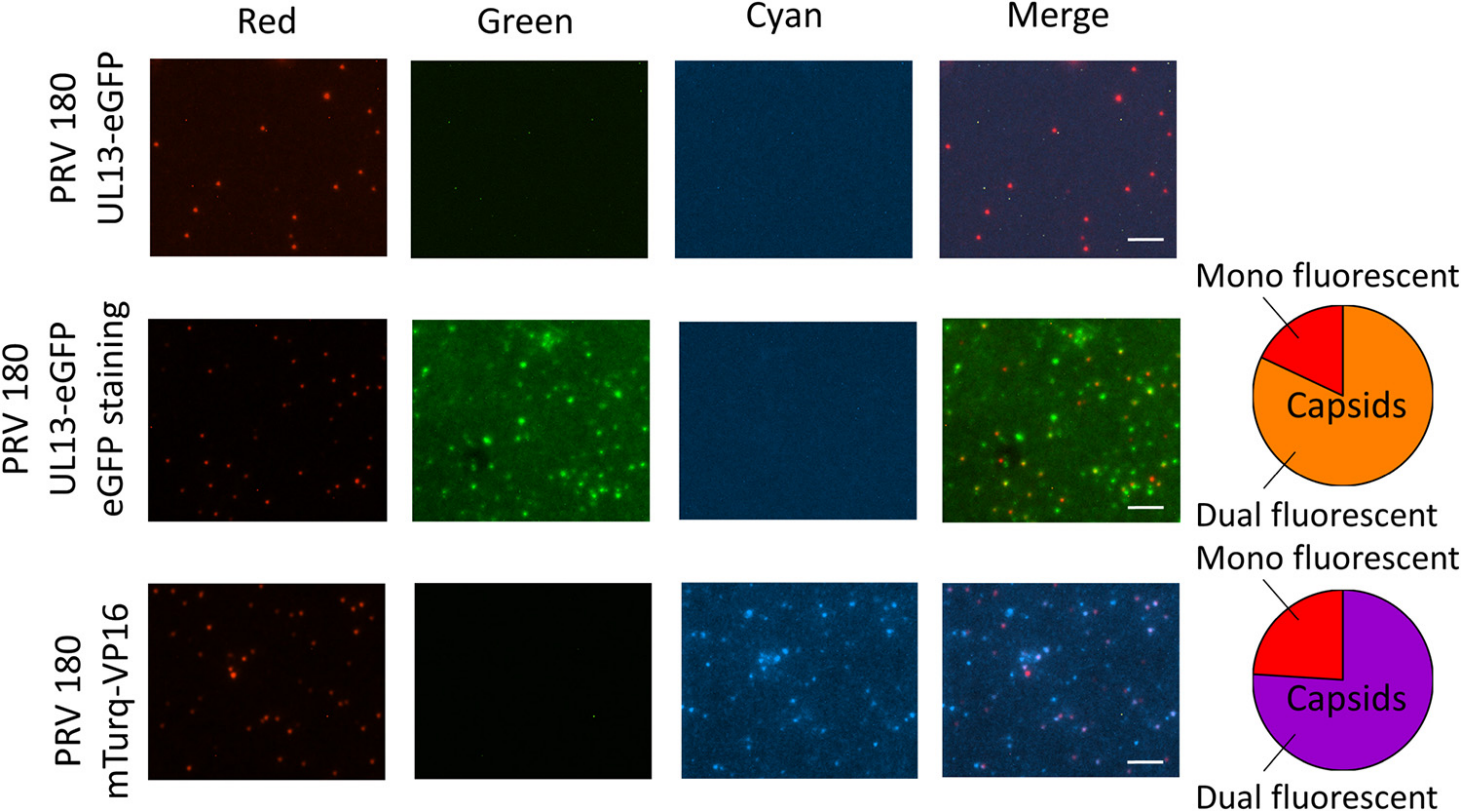
Validation of PRV 180 UL13-eGFP and PRV 180 mTurq-VP16 for live-cell imaging. Red particles (mRFP1-capsid) from purified viral stocks were analyzed for green (PRV 180 UL13-eGFP) and cyan (PRV 180 mTurq-VP16) fluorescence emission. PRV 180 UL13-eGFP particles were additionally stained with polyclonal rabbit anti-eGFP antibodies. Pie charts show the proportion of virions with structurally incorporated UL13 or VP16 (dual fluorescent) to capsids without UL13 or VP16 incorporation (mono fluorescent). Scale bars represent 10 μm.

Our newly constructed collection of dual fluorescent PRV mutants was used to track PRV capsids along with tegument proteins UL13 or VP16 during live axonal transport in chambered SCG neurons. To determine suitability of mutants in live-cell imaging, purified virus particles were first spotted onto coverslips and fluorescence was verified. We observed red as well as cyan fluorescent puncta in the PRV 180 mTurq-VP16 stock, but were unable to detect green fluorescent puncta in the PRV 180 UL13-eGFP stock (Fig. 4, upper panel). This result suggests that the amount of UL13-eGFP by itself was likely too low to emit detectable levels of green fluorescence. However, immunofluorescence staining of eGFP confirmed that 82% of monomer red fluorescent protein (mRFP1)-capsid-containing particles had incorporated UL13-eGFP (Fig. 4, middle panel). In the mTurq-VP16 virus stock, 76% of red puncta colocalized with cyan puncta, suggesting that the majority of mRFP1-capsid-containing particles in the virus stock had incorporated detectable mTurq-VP16 (Fig. 4, lower panel).

**FIG 5:**
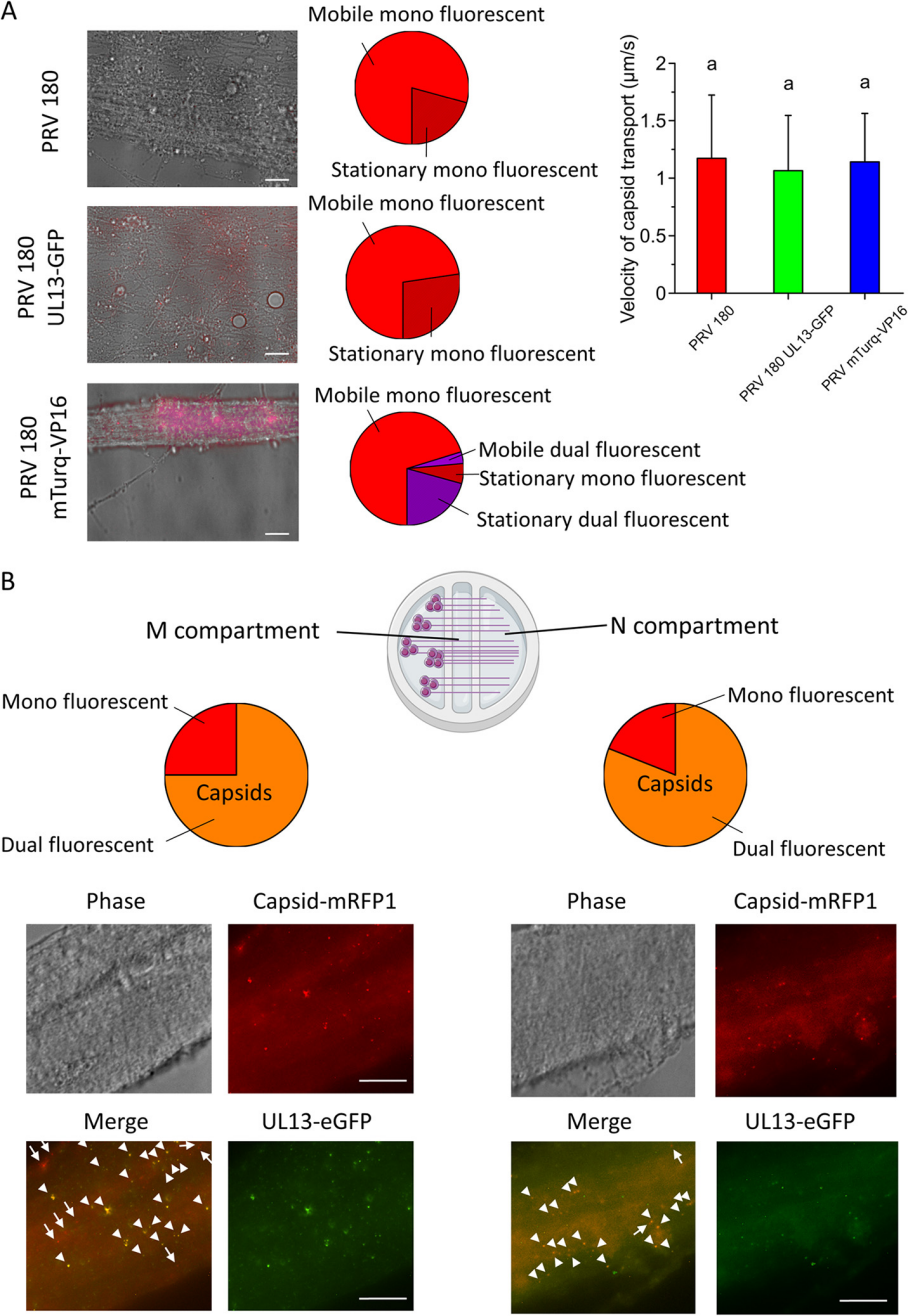
Axonal transport of UL13 and VP16 in compartmented SCG neurons. All scale bars represent 10 μm. (A) Live-cell imaging of PRV 180, PRV 180 UL13-eGFP, and PRV 180 mTurq-VP16 retrograde transport (see also Movie S1 to S3 in the supplemental material). Images show the merge of red, green, and phase of a Movie snapshot. Pie charts show the proportion of moving mono or dual fluorescent capsids to mono or dual fluorescent stationary capsids. Capsid velocities are given in the graph. Experiments were performed in triplicate. Different lower case letters represent significant differences (*P* < 0.05). (B) Immunofluorescence staining of eGFP in M (left) and N (right) compartment 2 hpi to track UL13 transport in combination with capsid transport. Pie charts show the proportion of dual fluorescent capsids to mono fluorescent capsids. Arrows and arrowheads point at mono and dual fluorescent capsids, respectively.

Next, we performed live-cell imaging of moving virus particles in SCG axons. Starting from 30 min after inoculation in the N compartment, mRFP1-labeled capsids of PRV 180 dual fluorescent strains were readily detected moving in a retrograde direction (i.e., toward the cell bodies) in SCG axons with dynamics similar to that of mono fluorescent PRV 180. Indeed, there was no significant difference in the percentage of moving capsids between PRV 180 (79.29 ± 7.00%), PRV 180 UL13-eGFP (72.67 ± 8.55%), and PRV 180 mTurq-VP16 (73.34 ± 10.87%) (pie charts in Fig. 5A). Further, the average velocity of capsid transport did not significantly differ between PRV 180 (1.17 ± 0.55 μm/s), PRV 180 UL13-eGFP (1.07 ± 0.48 μm/s), and PRV 180 mTurq-VP16 (1.14 ± 0.42 μm/s), as shown in Movie S1 to S3 in the supplemental material and the graph in Fig. 5A. These findings show that tagging UL13 or VP16 with a fluorophore does not influence transport dynamics of mRFP1-capsids of PRV 180. During retrograde transport, only 4.34 ± 3.22% of moving PRV 180 mTurq-VP16 capsids (red) emitted cyan fluorescence, while the majority of stationary particles (78.41 ± 6.98%) emitted red as well as cyan fluorescence.

Live-cell imaging of UL13 was not possible due to low green fluorescence emission. Therefore, axons in both the M and N compartments were fixed at 2 hpi and stained for eGFP to track UL13 localization (Fig. 5B). It should be noted that fixation caused a decrease in mRFP fluorescence and made it impossible to directly stain UL13 using our polyclonal rabbit serum. Still, staining eGFP enabled us to detect UL13-eGFP. Interestingly, the percentage of red capsid puncta colocalizing with green puncta was similar in the N compartment (75.51 ± 8.77%) as in the M compartment (80.69 ± 11.57%). These data indicate that UL13 is cotransported with PRV capsids during retrograde transport, while VP16 stays behind upon infection of neuronal axons.

### UL13 is involved indirectly in PRV escape from genome silencing in SCG neurons.

**FIG 6:**
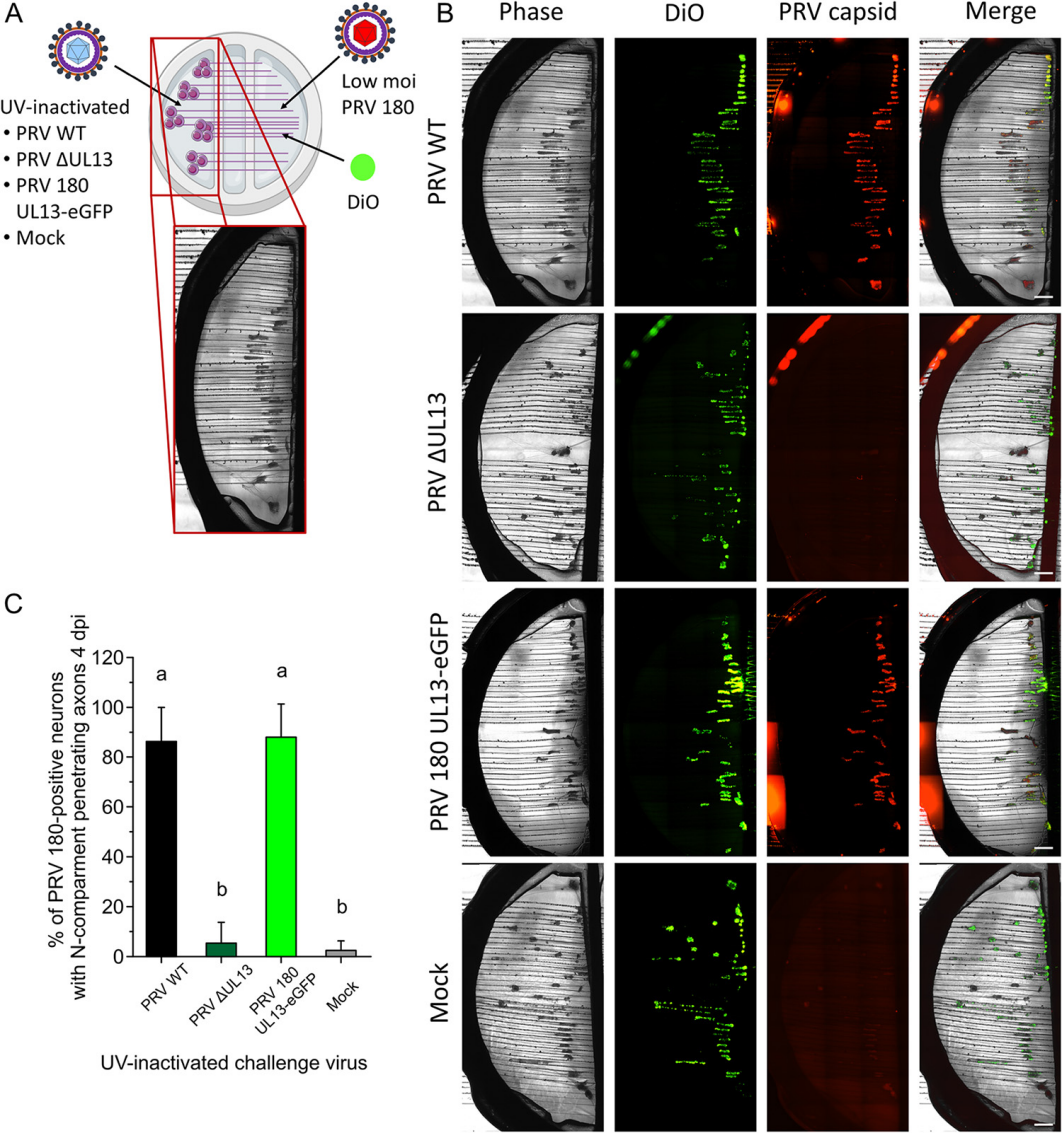
UL13 is important in the escape from genome silencing by PRV. (A) Schematic of complementation assay used to study escape from genome silencing in chambered SCG neurons. (B) Phase, fluorescent, and merged images of S compartments at 4 dpi using different stimuli in the S compartment. Dioctadecyloxacarbocyanine perchlorate (DiO) was added to the N compartment to visualize the number of neurons with N compartment-penetrating axons. Scale bars represent 1,000 μm. (C) The percentage of PRV capsid-positive neurons among the total number of neurons with N compartment-penetrating axons. Experiments were performed 5 times. Data are shown as means + SD and significant differences (*P* < 0.05) are indicated by different lower case letters.

Using a trichamber neuron culture system, we previously showed that delivery of a high dose of UV-treated or nucleocapsid-deficient PRV light (L) particles to neuronal cell bodies triggers escape from silencing of infectious PRV viruses applied to axons, suggesting that tegument proteins might be involved (4). To understand whether the viral kinase tegument protein UL13 plays a role, we applied 10^10^ genome copies of UV-inactivated PRV ΔUL13 virions to neuron cell bodies, while simultaneously inoculating axons with a low dose (10^2.5^ PFU) of red capsid-labeled PRV 180 (Fig. 6A). Dioctadecyloxacarbocyanine perchlorate (DiO) was added to the N compartment to identify neuronal cell bodies with axons that penetrate into the N compartment. Phase and fluorescent images are shown in Fig. 6B. As expected, 86.3 ± 13.6% of DiO-positive (i.e., connected with the N-compartment) neurons produced red fluorescence, corresponding to mRFP1-capsid proteins, 4 days after treatment with UV-inactivated PRV WT virions (Fig. 6C). This percentage was significantly (*P* < 0.05) higher compared to mock treatment (2.5 ± 3.8%) or treatment with UV-inactivated PRV ΔUL13 (5.3 ± 8.3%). Interestingly, we observed similar results when standardizing the inoculum of UV-inactivated viruses for PFU (10^5^ PFU), even though neuronal cell bodies are flooded with defective viral particles delivering virion proteins in the case of UV-inactivated PRV ΔUL13 compared to PRV WT. Fusion of UL13 to eGFP did not influence its function in escape from genome silencing, as indicated by the high percentage of connecting neurons that escaped from silencing upon treatment with UV-inactivated PRV 180 UL13-eGFP (88.0 ± 13.3%).

**FIG 7:**
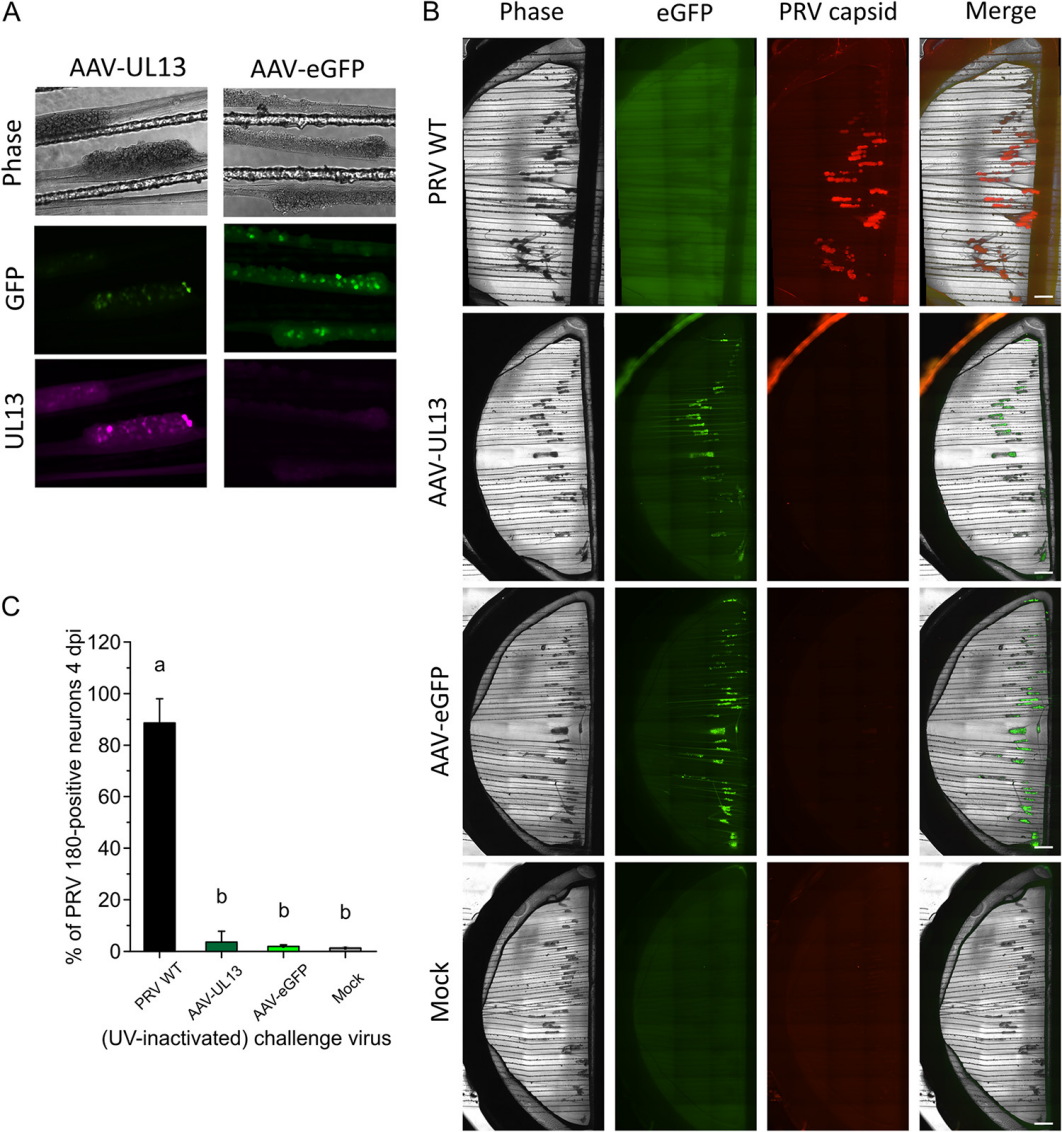
UL13 is not directly involved in the escape from genome silencing by PRV. (A) SCG neuronal bodies express eGFP and/or UL13 at 6 days upon inoculation by AAV vectors. (B) Phase, fluorescent, and merged images of S compartments at 4 dpi following AAV-induced UL13/eGFP expression in the S compartment. Scale bars represent 1,000 μm. (C) The percentage of PRV capsid-positive neurons among the total number of neurons. Experiments were performed 5 times. Data are shown as mean + SD and significant differences (*P* < 0.05) are indicated by different lower case letters.

To confirm the role of UL13 in PRV escape from genome silencing, we produced AAVs that induce the expression of UL13 and reporter protein eGFP linked by self-cleavable p2a in SCG neurons. AAVs expressing only p2a and eGFP functioned as controls. Neuronal cell bodies started to express eGFP at 2 to 3 dpi by AAVs, which accumulated over the following days. SCG neurons clearly express UL13 at 6 dpi with AAV-UL13, as demonstrated by immunofluorescence staining in Fig. 7A. Expression of UL13 did not influence cell viability, as analyzed using ReadyProbes cell viability imaging kit (Thermo Fisher Scientific). In contrast to UV-inactivated PRV WT, UL13-expressing neurons were not sufficient to facilitate PRV 180 genomes to escape from silencing after inoculation of axons (Fig. 7B and C). Instead, only 3.6 ± 4.2% of connecting neurons produced mRFP1-labeled capsid proteins in the presence of UL13 expression at 4 dpi, showing that UL13 does not play a direct role in the escape from genome silencing by PRV. Similarly, expression of eGFP or mock treatment of neurons did not induce escape from genome silencing by PRV. Together, these data show that UL13 plays an indirect role in the escape from genome silencing by PRV.

## DISCUSSION

Induction and escape from viral genome silencing in peripheral nervous system (PNS) neurons is a hallmark of alphaherpesvirus biology. The double-stranded DNA (dsDNA) from incoming virions either rapidly associates with histones, resulting in genome silencing, or is bound by RNA polymerase II to initiate the orderly cascade of viral gene transcription followed by virion production. Still, the exact molecular events that determine the fate of these incoming viral genomes are poorly understood. Using a compartmented complementation assay in trichambers, Koyuncu et al. (4) identified a new distinct molecular mechanism to start productive infection from quiescently destined PRV genomes: a rapid viral tegument-mediated route. For example, complementing neuronal cell bodies infected with UV-inactivated whole virions or light particles (containing tegument proteins but lacking nucleocapsids) facilitates productive axonal PRV 180 infection that was destined to be silenced. In the current study, we sought to identify the role of the viral kinase and tegument protein UL13 in the escape from PRV genome silencing in SCG neurons. This study required the construction of new PRV mutants. Although some PRV ΔUL13 mutants have been constructed in other PRV backgrounds, no PRV Becker ΔUL13 mutants without any other modifications had been constructed (16, 17). As the Becker strain efficiently induces escape from genome silencing, we constructed a UL13-deletion mutant in the PRV Becker background. Further, we also used the Becker mutant PRV 180 (which encodes the VP26-mRFP1 fusion protein that is incorporated into capsids) to construct mutants with UL13 fused to enhanced green fluorescence protein (eGFP). We also replaced the VP16 coding sequence with a hybrid gene encoding a fusion protein with VP16 fused to mTurquoise v2 (mTurq) as an internal control. We showed that homologous recombination-mediated gene replacement was highly efficient upon sgRNA/Cas9-nicking of target PRV DNA. Indeed, up to 83% of all progeny viruses formed fluorophore-positive plaques. This percentage is similar or even higher than previously described for the construction of PRV mutants through CRISPR/Cas9 (11, 12). However, these published studies did not screen selected sgRNAs for potential off-target effects. Nonspecific binding of sgRNAs to genomic regions outside the target sequence can result in unwanted mutations in these genes (18). Therefore, we used the CRISPOR algorithm to select sgRNAs without any potential off-target effects in the PRV genome (19).

Disruption of UL13 clearly reduced production of infectious virus, as PRV ΔUL13 produced a 10-fold lower viral titer compared to PRV WT at 18 hpi. This decrease was comparable to that previously reported for two PRV mutants carrying UL13 deletions (16, 20). Also, the infectivity and spread of PRV ΔUL13 in PK15 cells was decreased compared to PRV WT, as PRV ΔUL13 produced about 10-fold fewer plaques that were on average 1.5-fold smaller in diameter compared to PRV WT. This phenomenon has already been described for other alphaherpesviruses, such as HSV-1 lacking UL13 (21). It was suggested that HSV-1 tegument uncoating might be affected in the absence of UL13 through lack of phosphorylation of viral components, actin, or other cytoskeletal elements (22). In addition, HSV-2 UL13 was proposed to regulate nuclear egress by localized disruption of nuclear lamins (23). The latter hypothesis is consistent with the perinuclear localization we observed for UL13, but especially for the fusion protein UL13-eGFP. These data indicate that during virus propagation, UL13 accumulates close to nuclear lamins and might be involved in nuclear egress of PRV. In the absence of UL13, nuclear egress of PRV and thus virus propagation might be affected. Still, the exact role of UL13 in the infectivity and spread of PRV remains enigmatic.

As described for HSV-1 strain KVP26mRFP1 expressing a VP26 fusion with mRFP, the replication of PRV 180 was decreased compared to the parental strain. This difference might be caused by the steric hindrance of mRFP or by a destabilization effect of fusion capsid proteins during capsid formation. However, unlike disrupting UL13, fusing the kinase to eGFP did not affect virus propagation kinetics, infectivity, or spread. Indeed, the viral titers, number of plaques, and plaque diameters of PRV 180 UL13-eGFP did not significantly differ from those of its parental strain, PRV 180. These data also suggest that tagging UL13 with eGFP does not influence its (kinase) activity, as PRV ΔUL13 induced smaller plaques, while PRV UL13-eGFP did not. The fact that PRV UL13-eGFP was still able to induce escape from silencing by PRV virions destined for quiescence, while PRV ΔUL13 was not, corroborates this hypothesis. Similarly, fusing mTurq to the N- or C-terminal end of VP16 did not decrease its infectivity, as described previously (9). Finally, fusing UL13 to eGFP did not affect velocities of capsid trafficking in SCG axons, as capsid transport velocities were similar for all fusion mutants and the parental virus. These velocities of capsid transport also corresponded to those previously published for PRV 180 in chicken embryo dorsal root ganglion neurons (DRGs) (9).

Further, our initial hypothesis was that during retrograde viral transport in axons, UL13 moves separately from PRV nucleocapsids, similar to what was described for VP16 (9). In our hypothesis, PRV capsids would arrive at the nuclear pore before UL13, preventing the tegument kinase from activating PRV genome transcription and thus favoring UL13 quiescence. However, we observed that while VP16 separates from capsids and stays behind upon axonal inoculation of SCG neuronal axons, at least some UL13 copies are cotransported with capsids during retrograde transport in axons. Although we were unable to track live transport of UL13 using eGFP fluorescence, we were able to detect UL13-eGFP by immunofluorescence staining. In this way we observed that red capsids arriving in the M compartment still contain UL13-eGFP. We were unable to visualize UL13-eGFP directly with antibodies to UL13, presumably because of the small amount of UL13 incorporated in the virion. Indeed, even mass spectrometry was not sensitive enough to detect UL13 in PRV virions (24). Still, Western blot and immunofluorescence staining of purified PRV virions demonstrated that UL13 is structurally incorporated into PRV virions. As a kinase enzyme, UL13 can be reused in multiple cycles, which may explain why low concentrations of UL13 are incorporated into virions. Similarly, Us3, another important serine/threonine-protein kinase, is also cotransported with PRV capsids during retrograde axonal infection (9). It was suggested that these viral kinases might facilitate PRV capsid transport by stabilizing membrane-capsid and microtubule-capsid interactions.

The fact that UL13 is cotransported with PRV capsids does not exclude its function in escape from genome silencing by PRV. Indeed, we demonstrated that escape from genome silencing indirectly requires viral tegument protein UL13. Following infection of neuronal cell bodies with UV-inactivated PRV virions devoid of UL13, axonal PRV 180 infection was silenced. In contrast, cell body infections with UV-inactivated PRV wild type virions enabled a productive infection by axonally transported PRV 180 particles. However, UL13 alone expressed in cell bodies from AAV vectors was not sufficient to induce PRV genome silencing. It should be noted that we were technically unable to verify the kinase activity of AAV-expressed UL13, as no such tests were then readily available. Still, our findings from the compartmentalization assays and cotransport analyses suggest that UL13 is involved in PRV escape from genome silencing through indirect mechanisms. We propose that another viral tegument protein, yet to be identified, primes SCG neurons for productive axonal infection upon phosphorylation by UL13. In the absence of UL13, this tegument protein is not phosphorylated and thus remains inactive. For HSV, UL13 has been suggested to phosphorylate glycoprotein E and I, UL41, Us3, ICP0, VP22, and ICP22 (22, 25–30). Glycoproteins E and I are embedded in the viral envelope and likely remain at the plasma and/or endocytic membranes during virion entry. UL41 is a virion host shutoff endoribonuclease with no apparent effect on viral gene transcription, and the role of Us3 and EP0 (orthologue of HSV ICP0) in PRV escape from silencing have already been ruled out (4, 31). Therefore, these proteins are unlikely to facilitate productive PRV infection in the compartmentalization assay. In contrast, tegument proteins VP22 and ICP22 are interesting candidates for further research. Major tegument protein VP22 of HSV-1 inhibits nucleosome deposition on DNA by binding to TAF-I (template-activating factor I) and thereby activates viral gene transcription (32). It may be that nonphosphorylated PRV VP22 is unable to inhibit nucleosome deposition on incoming PRV genomes and, thus, trigger escape from silencing. PRV ICP22 is a tegument protein important for proper PRV gene expression (33). Interestingly, HSV-1 ICP22 is extensively phosphorylated and mediates, in conjunction with UL13, the phosphorylation of RNA polymerase II (7, 30, 34). Perhaps this interplay between UL13 and ICP22 is also necessary to induce escape from silencing. Finally, it is also possible that multiple proteins need to be activated by UL13 phosphorylation in order to prevent genome silencing. Alternatively, UL13 might act in parallel with another tegument protein and both might be required for efficient escape from genome silencing. Future studies, including the addition of UV-inactivated PRV ΔUL13 to AAV-UL13 transduced cell bodies in *trans* and characterization of viral protein phosphorylation profiles, will help to uncover the role of these tegument proteins.

In this report, we used CRISPR/Cas9-constructed PRV mutants and complementing AAV transduction to show that UL13 is indirectly involved in PRV escape from genome silencing in neurons. Accordingly, it may act through phosphorylation of other tegument proteins (e.g., VP22 or ICP22). Further, we showed that, unlike VP16, UL13 remains with PRV capsids after axonal entry and may mediate interactions between PRV capsids and cellular microtubules or membranes. These new insights in alphaherpesvirus escape from quiescence will forward the development of efficient antiviral therapies, a significant aspect of medical research on herpesvirus-induced diseases.

## MATERIALS AND METHODS

### Cells.

**Cell lines.** Porcine kidney epithelial cells (PK15; ATCC, Manassas, VA, USA) were used to produce and titer PRV stocks. Human embryonic kidney cells expressing large T antigen (HEK293T cells; ATCC) were used to produce CRISPR lentiviruses, PRV mutants, and adeno-associated viruses (AAVs). All cells were maintained in Dulbecco's modified Eagle medium (DMEM) supplemented with 10% fetal bovine serum (FBS), 1% penicillin, and streptomycin (DMEM complete medium).

**Primary neurons.** Superior cervical ganglia (SCGs) were isolated from embryonic day 17 Sprague-Dawley rat embryos (Hilltop Labs, Scottdale, PA, USA) as previously described (35). SCG neurons were cultivated either dissociated in plain dishes or compartmented in trichamber-mounted dishes. Plastic tissue culture dishes (35 mm, Falcon) or optical plastic dishes (Ibidi) were coated with 500 μg/ml of poly-DL-ornithine (Sigma-Aldrich, St. Louis, MO, USA) and 10 μg/ml of natural mouse laminin (Invitrogen). Trichambers were installed onto these dishes for compartmentation of neurons and axons as previously described (5). Neurons were cultured for a minimum of 4 weeks prior to experiments.

**Viruses.** PRV Becker is a wild-type (PRV WT) strain commonly used as background for PRV mutants (36). PRV 180 expresses a monomeric red fluorescent protein (mRFP)-VP26 fusion protein in a PRV Becker background (15). PRV ΔUL13, PRV 180 eGFP-UL13, PRV 180 mTurq-VP16, and PRV 180 VP16-mTurq viruses were constructed using CRISPR/Cas9-mediated gene replacement, as described below.

### Plasmids and sgRNA selection.

Synthesized oligonucleotide primers (Integrated DNA Technologies, Coralville, IA, USA) corresponding to separate targets were cloned into an EspI-digested (New England BioLabs [NEB], Ipswich, MA, USA) LentiCRISPR v2 plasmid (gift from Feng Zhang; Addgene plasmid number 52961). Guide sequences were selected using CRISPOR by containing minimal mismatches to any human or PRV genomic sequences, while maximizing the presumable on-target effect (37). Selected target sites and protospacer adjustment motif (PAM) sequences are shown in Table 1. There were no potential off-target regions in the PRV genome, as determined using CRISPOR (http://crispor.tefor.net/) (19).

Donor plasmids were constructed using the HiFi DNA assembly method (NEB), as described below. All donor plasmids consisted of a pcDNA3-eGFP backbone (gift from Doug Golenbock; Addgene plasmid number 13031) with an upfront and downstream homology arm with eGFP or mTurquoise in between (Fig. 1B). Each set consisted of four fragments, which were PCR-amplified from purified PRV DNA (homology arms), pcDNA3-eGFP (vector backbone and eGFP), or pcDNA3-mTurq (mTurquoise v2) using Q5 high-fidelity polymerase (NEB) and specific primer pairs shown in Table 3 (Integrated DNA Technologies). Silent mutations (underlined) were introduced in PAM sequences of PRV 180 fusion mutants.

**TABLE 3:** Fragment and primer design for HiFi DNA assembly

Donor plasmid	Name	Length (bp)	Primer design (5′–3′)^*a*^	
			Forward	Reverse
UL13 deletion donor plasmid	Vector backbone	5,420	cgggttcctg-TCTAGAGGGCCCTATTCTATAG	ctttgccatc-TGTGATGGATATCTGCAG
	Upfront homology arm	609	atccatcaca-GATGGCAAAGTTGAAAAAGCGGGC	cggccgccag-TCACGCCTCCTCCGCCTC
	eGFP	756	ggaggcgtga-CTGGCGGCCGCTCGAGAT	gcgccgccat-TTACTTGTACAGCTCGTCCATGCCG
	Downstream homology arm	875	gtacaagtaa-ATGGCGGCGCTCGTTTTGC	gccctctaga-CAGGAACCCGCGCAGCGT
UL13-eGFP fusion donor plasmid	Vector backbone	5,420	ggaggagctg-TCTAGAGGGCCCTATTCTATAG	cgccataaag-TGTGATGGATATCTGCAG
	Upfront homology arm	650	atccatcaca-CTTTATGGCGGCCAAACAGG	tgctcaccat-GAGCGTCTTCACGGCCAC
	eGFP	737	gaagacgctc-ATGGTGAGCAAGGGCGAG	agccggcgcg-CTTGTACAGCTCGTCCATGC
	Downstream homology arm	764	gctgtacaag-CGCGCCGGCTTCGG**A**CAC	gccctctaga-CAGCTCCTCCTCGAGGATGTCCCC
mTurq-VP16 fusion donor plasmid	Vector backbone	5,420	gtcgaggagc-TCTAGAGGGCCCTATTCTATAG	tcgagctgga-TGTGATGGATATCTGCAG
	Upfront homology arm	821	atccatcaca-TCCAGCTCGAGAAAGACCCGG	tgctcaccat-CCTCACCGACCCCCCCAC
	mTurq	737	gtcggtgagg-ATGGTGAGCAAGGGCGAG	cgtcgcgcat-CTTGTACAGCTCGTCCATGC
	Downstream homology arm	873	gctgtacaag-ATGCGCGACGAGGAGTGCGTGGTCGCGTTCGACGA**A**	gccctctaga-GCTCCTCGACCAGGTCGG
VP16-mTurq fusion donor plasmid	Vector backbone	5,420	gactacctgt-TCTAGAGGGCCCTATTCTATAG	aagaagcgct-TGTGATGGATATCTGCAG
	Upfront homology arm	874	atccatcaca-AGCGCTTCTTCGTGTCCAC	tggcgaccgg-CATCTCAAACATCC**T**GTTGAG
	mTurq	752	gtttgagatg-CCGGTCGCCACCATGGTG	gcgcgcggcg-TTACTTGTACAGCTCGTCCATGCC
	Downstream homology arm	595	gtacaagtaa-CGCCGCGCGCGGTCGGAT	gccctctaga-ACAGGTAGTCCACGTCGGCGGG
AAV-eGFP	Vector backbone	4,325	cggtccttga-AAGCTTATCGATAATCAACCTCTGG	tgctcaccat-GCTAGCGGATCTGACGGTTC
	eGFP	641	atccgctagc-ATGGTGAGCAAGGGCGAG	ctttgctcag-GGCGGACTGGGTGCTCAG
	eGFP-p2a	185	ccagtccgcc-CTGAGCAAAGACCCCAAC	cgataagctt-TCAAGGACCGGGGTTTTC
AAV-UL13	Vector backbone	4,325	cgctgcctga-AAGCTTATCGATAATCAACCTCTGG	tgctcaccat-GCTAGCGGATCTGACGGTTC
	eGFP	641	atccgctagc-ATGGTGAGCAAGGGCGAG	ctttgctcag-GGCGGACTGGGTGCTCAG
	eGFP-p2a	182	ccagtccgcc-CTGAGCAAAGACCCCAAC	cagcagccat-AGGACCGGGGTTTTCTTC
	UL13	1,217	ccccggtcct-ATGGCTGCTGGAGGAGGC	cgataagctt-TCAGGCAGCGAGTTCGGC

a PAM, protospacer adjustment motif. Silent mutations (bold) were introduced in PAM sequences of PRV 180 fusion mutants. Lowercase letters represent the overlapping part of the primer, while the uppercase letters represent the annealing part of the primer.

The backbones of AAV plasmids (CMV-eGFP-p2a-WPRE-SV40pA or CMV-eGFP-p2a-UL13-WPRE-SV40pA) were generated by cloning. Purified viral DNA, a pAAV-mTurq-p2a-WPRE-SV40pA (Engel lab) and pcDNA-eGFP vector were modified by PCR and assembled using HiFi DNA assembly (NEB). Specific fragments and primer pairs are shown in Table 3.

### Lentivirus production and transduction.

Lentiviruses were produced in HEK293T cells by cotransfecting LentiCRISPRv2 plasmids containing separate targets with packaging plasmids pMD2.G and psPAX2 using polyethylenimine (PEI) reagent (VWR International, Radnor, PA, USA) at a DNA:PEI ratio of 1:3 (wt/wt) following a chloroquine hydrochloride (25 μM; Sigma-Aldrich) preincubation step. Fifty-six hours posttransfection, the cell supernatant was collected, centrifuged at 900 × *g*, clarified by passing through a 0.45-μm polyethersulfone (PES) filter, and stored at –80°C. Lentiviral titers were determined using the QuickTiter lentivirus titer kit according to the manufacturer’s instructions (Cell Biolabs, San Diego, CA, USA).

Fresh HEK293T cells were transduced for 48 h with CRISPR lentiviruses at an MOI of 2.5 in the presence of 10 μg/ml polybrene (Sigma-Aldrich). After 48 h, medium was replaced daily by fresh DMEM medium containing 10 μg/ml puromycin for 5 days. Stably transfected cells were then expanded and maintained in DMEM medium containing 5 μg/ml puromycin. Monoclonal cell lines (HEK293T-UL13sgRNA, HEK293T-VP16sgRNA1, and HEK293T-VP16sgRNA2) were generated by limiting dilution in order to maintain a stable expression of sgRNAs and Cas9. Briefly, 100 μl of a cell suspension of 5 cells/ml was seeded into each well of a 96-well plate. After 7 days of incubation, the wells were visually inspected for colony formation. Wells harboring only 1 colony were selected, while those without any or with 2 or more colonies were discarded. After full expansion, Cas9 expression was evaluated using the Cas9 enzyme-linked immunosorbent assay (ELISA) kit of Cell Biolabs according to the manufacturer’s instructions. Monoclonal cell lines expressing between 150 and 200 ng/ml Cas9 were selected for downstream experiments.

### Adeno-associated virus production.

AAV plasmids containing either CMV-eGFP-p2a-WPRE-SV40pA or CMV-eGFP-p2a-UL13-WPRE-SV40pA were packaged into AAV-PHP.eB capsids (gift from Viviana Gradinaru, Addgene plasmid number 103005) at the Princeton Neuroscience Institute Viral Core Facility (38). AAV particles were purified by iodixanol step gradient followed by column ultrafiltration as previously described (39, 40). Infectious viral genomes were measured by Taq-Man quantitative PCR (qPCR).

### CRISPR/Cas9-mediated gene editing.

HEK293T-UL13sgRNA, HEK293T-VP16sgRNA1, and HEK293T-VP16sgRNA2 cells were pretreated with chloroquine hydrochloride prior to transfection with mock plasmids or donor plasmids using PEI reagent as described above. After 24 h, cells were inoculated with either mock, PRV WT, or PRV 180 at an MOI of 1. Cells were further maintained in DMEM complete medium supplemented with 10 μM SCR7 and 10 μM RS-1 (Sigma-Aldrich) to inhibit nonhomologous end joining and enhance homologous recombination, respectively. Forty-eight hours postinoculation, cells and supernatant were harvested, pooled, and stored at –80°C. Viral stocks were titrated on PK15 cells using classic plaque assay and screened for eGFP or mTurquoise expression by immunofluorescence microscopy. Fluorescent plaques were subjected to 3 rounds of plaque purification before propagating viral stocks on PK15 cells.

### Quantitative PCR analysis and sequencing.

Viral DNA was purified from different PRV stocks using the QIAamp MinElute virus spin kit (Qiagen, Hilden, Germany) according to the manufacturer’s instructions. UL13 and VP16 gene regions were amplified using primer pairs UL13 seq and VP16 seq (Table 2) using Q5 high fidelity polymerase (NEB) in the presence of 4% dimethyl sulfoxide (DMSO) (Sigma-Aldrich) and 2.5 M betaine (Sigma-Aldrich). Sequences were confirmed by Sanger sequencing (Genewiz, South Plainfield, NJ, USA).

Genome copies per ml were determined for each virus stock by means of quantitative PCR (qPCR). Virus stocks were first treated with 100 U of DNase I for 30 min at 37°C (Thermo Fisher) followed by inactivation for 10 min at 80°C. Samples were then digested with proteinase K (NEB) in 0.5% Tween 20 for 60 min at 55°C, followed by inactivation for 10 min at 95°C. Viral genomic DNA was quantified using UL54-specific primers (Table 2), as previously published (41). A serial dilution of purified whole genome of PRV Becker virions functioned as a standard. Triplicate reaction mixtures were prepared using a KAPA SYBR FAST qPCR kit (Sigma-Aldrich). Each experiment was performed in duplicates. The qPCR was performed with an Eppendorf RealPlex Mastercycler with the following amplification conditions: preincubation at 95° for 2 min with 40 cycles of denaturation (5 s at 95°C), annealing (20 s at 55°C), and extension (10 s at 72°C). The threshold cycle (*C_T_*) values were calculated using Mastercycler EP RealPlex 2.2 software. Sample *C_T_* values were plotted against standard dilution values to determine exact genomic DNA concentrations. Finally, DNA concentrations were converted into viral genome copies/ml based on the total PRV genome size (141,113 bp).

### Virus purification.

Culture supernatants of PRV-infected PK15 cells were clarified by centrifugation at 40,000 × *g* for 30 min at 4°C. The virion pellet was pooled onto a discontinuous OptiPrep gradient (Sigma-Aldrich) containing 10 to 30% (wt/vol) of iodixanol and centrifuged at 100,000 × *g* for 2.5 h at 4°C. After centrifugation, purified opalescent virion bands were harvested at the interface of the 15% and 20% layers. Virion bands were pooled in HNE buffer (5 mM HEPES, 150 mM NaCl, 0.1 mM EDTA, pH 7.4) by the use of a 50K filter device (Millipore Corporation, Bedford, MA, USA).

### Compartmented complementation assay in primary neurons.

SCG neurons were infected by adding a low MOI (10^2.5^ PFU) PRV 180 to the axonal (N) compartment to enable genome silencing after axonal transport. UV-inactivated PRV WT or mutants (10^10^ genome copies ∼10^5–6^ PFU) were added at the same time into the S compartment. We chose to standardize for genome copies instead of PFU, as PRV mutant stocks might contain more noninfectious virus particles compared to PRV WT. These defective particles might still be capable of delivering their content (e.g., tegument proteins) to neurons, thereby biasing the results. AAVs were added 3 days prior to PRV inoculation to ensure stable UL13 and/or eGFP expression, but no overexpression, prior to inoculation.

### Virus plaque assay.

PK15 cells were grown to confluence in 6-well dishes prior to inoculation with 10^6^ genome copies (∼10 to 100 PFU) of PRV WT or PRV mutants for 1 h at 37°C. Next, cells were covered with a solution containing 50% 2× MEM (Thermo Fisher Scientific) and 50% 1.88% carboxymethylcellulose (Sigma-Aldrich). Forty-eight hours postinoculation (hpi), medium was removed, and cells were fixed in ice-cold methanol for 20 min at −20°C. To visualize virus plaques and/or single infected cells, viral immediate early (IE) 180 protein was stained for immunofluorescence as described below using a rabbit polyclonal anti-IE180 protein antibody and Alexa Fluor 488-conjugated goat anti-rabbit antibodies (42).

### Viral growth kinetics.

PK15 cells were grown to confluence in 24-well dishes and dissociated SCGs were cultivated for 4 weeks on optical plastic dishes prior to inoculation. Cells were inoculated with PRV WT or mutants at an MOI of 1 for 1 h at 37°C. Next, cells were briefly exposed to 40 mM citrate buffer (pH 3) to inactivate any free virus particles left from the inoculum that had not infected. After 3 washing steps with DMEM, cells were further incubated with complete medium at 37°C. At the indicated time points, supernatants were collected for virus titration and cells were fixed in ice-cold methanol (20 min at −20°C) for immunofluorescence staining.

### Virus titration.

PRV titrations were conducted on PK15 cells, which were incubated at 37°C for 7 days. Titers were expressed as 50% tissue culture infective dose (TCID_50_).

### Immunofluorescence staining.

**Purified virus particles.** For immunofluorescence staining of purified virus particles, 1 μl of purified virus stocks was spotted onto glass coverslips and left to adsorb to the glass for 15 min at 37°C. Particles were permeabilized in 0.1% Triton X-100 followed by 1 h staining at 37°C with primary antibodies (rabbit anti-UL13 antibody, produced in-house, rabbit anti-VP16 antibody, produced by GenScript [Piscataway, NJ, USA] or isotype control rabbit IgGs against rabies virus nucleoprotein [43]) diluted in phosphate-buffered saline (PBS) with 10% normal goat serum (NGS) and Alexa Fluor 647-conjugated secondary antibody (Thermo Fisher Scientific). Particles were washed 3 times with PBS for 5 min per wash prior to and after secondary antibody staining.

**Viral growth kinetics in PK15 cells and SCG neurons.** For immunofluorescence staining of infected PK15 cells and dissociated SCGs, cells were fixed in methanol at −20°C for 20 min at the indicated times after infection. Primary antibodies (rabbit polyclonal anti-UL13, rabbit polyclonal anti-VP16, rabbit polyclonal anti-VP26, mouse monoclonal anti-Us3, and isotype controls [44]) were diluted in 10% NGS-PBS and added to cells for 1 h at 37°C followed by incubation of 1 h at 37°C with Alexa Fluor 488 (green)-, 594 (red)-, or 647 (magenta)-conjugated secondary antibodies. During the last 10 min of secondary antibody staining, DAPI (4′,6-diamidino-2-phenylindole) was added to cells to stain cell nuclei.

**Chambered SCG neurons.** For immunofluorescence staining of chambered SCGs during virus trafficking experiments, cells were fixed in 1% paraformaldehyde (PFA) at room temperature (RT) for 10 min, permeabilized in 0.1% Triton X-100 for 2 min at RT. Staining was performed with primary polyclonal rabbit anti-eGFP antibodies (Invitrogen) or isotype control antibodies and secondary Alexa Fluor 488-conjugated antibodies, as described above.

### Live-cell imaging.

All imaging was done using a previously described Nikon Ti-E inverted epifluorescence microscope (45).

**Virus particle trafficking.** Virus particle trafficking was assessed using live-cell imaging as described previously (46). Briefly, chambered SCGs grown onto Ibidi glass dishes were placed inside a stage top incubator system at 37°C and 5% (vol/vol) CO_2_ (Live Cell Instrument/Quorum Scientific), prior to inoculation with 10^6^ PFU of virus. Movies were acquired either with phase, green, red, or cyan fluorescence filters using a 60× Plan Fluor Ph3 objective (Nikon) and an iXon 895 back-thinned electron multiplying charge-coupled device (EM-CCD) camera (Andor, Belfast, Northern Ireland). The imaging window was maintained at a fixed height of 100 pixels and a width of 300 pixels. The fluorescence exposure was set to 200 ms, with the EM Gain filter set to 300. The acquisition rate was approximately 4 frames per second, on average. For the dual fluorescent movies, the exposure was set to 300 ms and the acquisition rate was approximately 1 frame per second. Movies were created and virus particles were manually tracked in infected axons using ImageJ software (National Institutes of Health, Bethesda, MD, USA).

**Escape from silencing.** Escape from silencing was assessed on tiled images of the entire S compartment. These images were captured using the Nikon NIS Elements software, a Cool Snap ES2 camera (Photometrics), and a 4× magnification objective (Nikon). Color thresholds were set manually, prior to converting images to 8-bit gray-scale images using ImageJ. Next, the relative amount of red fluorescence (viral production) was divided by the amount of green fluorescence (only those neurons that project their axons to the N compartment) to determine the percentage of susceptible neurons that escaped from silencing. Since the majority of neurons sent axons through the N compartment (>90%), only the percentage of red fluorescence was monitored (viral production) upon AAV inoculation.

**Colocalization image analysis.** Colocalization of red (capsids) and green/cyan (UL13/VP16) puncta was verified by image analysis using ImageJ. Briefly, RGB images were converted to 8-bit images and subjected to conservative thresholding. Next, binary image multiplication was used to determine the overlap of puncta. The results of 5 microscopic fields were added to obtain a total value.

Colocalization of UL13 with eGFP, and VP16 with mTurquoise, respectively, in infected cells was verified by image analysis using ImageJ. Briefly, RGB images were converted to 8-bit images. Next, binary image subtraction was used to determine the overlap of colors.

### Western blot analysis.

Virion protein content of newly constructed PRV mutants was characterized by Western blotting. For this, 10^12^ genome copies of purified virus stocks were mixed with 5× Laemmli buffer and heated at 95°C for 5 min. Samples were then loaded onto 10% NuPAGE BisTris gels (Invitrogen) and run for 15 min at 100V and 45 min at 200V in MOPS (morpholinepropanesulfonic acid) buffer. Proteins were transferred to nitrocellulose membranes using semidry transfer at 18V for 90 min. Next, membranes were washed 2 times with ultrapure water for 2 min and incubated in antigen pretreatment solution (Invitrogen) for 10 min at RT. After 5 rinses with ultrapure water, membranes were incubated in 5% nonfat dry milk in phosphate-buffered saline supplemented with 0.1% Tween 20 (PBST) solution for 1 h at RT. Following three 5-min washing steps in PBST, membranes were stained with primary antibodies diluted 1:1,000 in primary antibody diluent (Invitrogen) overnight at 4°C. Secondary antibodies (goat anti-mouse or anti-rabbit antibodies conjugated to horseradish peroxidase [HRP] [Thermo Fisher Scientific]) were diluted 1:20,000 in 1% milk-PBST solution and added to the membranes for 30 min at RT after and prior to a thorough washing step (4 times 5 min washing in PBST). Membranes were incubated with chemiluminescent substrates (Supersignal Dura, Thermo Fisher scientific). Protein bands were visualized by exposure on HyBlot CL (Denville Scientific) blue X-ray films. Primary antibodies used for Western blot were anti-VP5 mouse monoclonal antibody (MAb) (gift of H. J. Rziha, Federal Research Center for Viruses Diseases for Animals, Tubingen, Germany), anti-Us3 mouse MAb (44), and anti-UL13 polyclonal rabbit sera (produced in-house). VP5 (capsid) protein functioned as an internal loading control to determine the relative amount of proteins present in different purified virus stocks. This was determined through plot profiling in ImageJ.

### Statistical analyses.

Significant differences (*P* < 0.05) between different CRISPR gene editing methods or between mock-inoculated and different PRV mutant-inoculated cells were identified by analysis of variance (ANOVA) followed by a Tukey *post hoc* test. If homoscedasticity of the variables was not met, as assessed by Levene’s test, the data were log transformed prior to ANOVA. The normality of the residuals was verified using the Shapiro-Wilk test. If the variables remained heteroscedastic or normality was not met after log transformation, a Kruskal-Wallis test followed by a Mann-Whitney *post hoc* test were performed. All analyses were conducted in IBM SPSS Statistics for Windows, version 25.0 (IBM Corp., Armonk, NY, USA).

### Ethics statement.

All animal work was performed in accordance with the Princeton Institutional Animal Care and Use Committee (protocols 1947–16). Princeton personnel are required to adhere to applicable federal, state, local, and institutional laws and policies governing animal research, including the Animal Welfare Act and Regulations (AWA), the Public Health Service Policy on Humane Care and Use of Laboratory Animals, the Principles for the Utilization and Care of Vertebrate Animals Used in Testing, Research and Training, and the Health Research Extension Act of 1985.

### Data availability statement.

The raw data supporting the conclusions of the manuscript will be made available by the authors, without undue reservation, to any qualified researcher.

## Supplementary Material

Supplemental file 1

Supplemental file 2

Supplemental file 3

Supplemental file 4
