# Supplementary material for: CRISPR/Cas9-Constructed Pseudorabies Virus Mutants Reveal the Importance of UL13 in Alphaherpesvirus Escape from Genome Silencing
Source: J Virol. 2021 Feb 24;95(6):e02286-20. doi: 10.1128/JVI.02286-20 (PMC8094956; doi:10.1128/JVI.02286-20)
Supplement: Supplemental file 4 [file JVI.02286-20-s0004.pdf]

Movie legends

CRISPR/Cas9-constructed pseudorabies virus mutants reveal the importance of UL13 in alphaherpesvirus escape from genome silencing

Jolien Van Cleemput[1], Orkide O. Koyuncu[1], Kathlyn Laval[1], Esteban A. Engel[2], Lynn W. Enquist[1#]

[1]Department of Molecular Biology, Princeton University, Washington Road, Princeton, NJ 08544, USA

[2]Princeton Neuroscience Institute, Princeton University, Princeton, NJ 08544, USA

Movie 1

Microscopy movie of PRV 180 retrograde transport in SCG axons (merge of red channel). Red puncta represent mRFP-capsids of PRV. Merge of red and phase channel and scale bare are shown in Fig. 5A.

Movie 2

Microscopy movie of PRV 180 UL13-eGFP retrograde transport in SCG axons (merge of red and green channel). Red puncta represent mRFP-capsids of PRV. Merge of red, green and phase channel and scale bare are shown in Fig. 5A.

Movie 3

Microscopy movie of PRV 180 mTurq-VP16 retrograde transport in SCG axons (merge of red and cyan channel). Red puncta represent mRFP-capsids of PRV. Cyan puncta represent mTurq-VP16 of PRV. Merge of red, cyan and phase channel and scale bare are shown in Fig. 5A.
